# Bone Health Status, Muscular Strength and Power, and Aerobic and Anaerobic Capacities of Malaysian Male Athletes Involved in Sports with Different Mechanical Loading on Bones

**DOI:** 10.21315/mjms2022.29.3.6

**Published:** 2022-06-28

**Authors:** Norsuriani Samsudin, Foong Kiew Ooi, Chee Keong Chen

**Affiliations:** Exercise and Sports Science Programme, School of Health Sciences, Universiti Sains Malaysia, Kelantan, Malaysia

**Keywords:** aerobic capacity, anaerobic capacity, bone, muscular strength

## Abstract

**Background:**

Physical activity is beneficial for bone health. Bones respond and adapt to applied loads that can vary among physical activity. This study investigated differences in bone health status, muscular performance, and aerobic and anaerobic capacities of young male Malaysian athletes competing at the state level.

**Methods:**

A total of 44 participants (age: 17.1 ± 1.6 years old) were randomly divided into sedentary control, weightlifting, cycling or squash groups. The bone speed of sound (SOS), muscular performance, maximal oxygen uptake (VO_2max_) and anaerobic capacities of the participants were measured.

**Results:**

All athletes exhibited significantly higher tibial and radial bone SOS (*P* < 0.01) values than the sedentary group. Weightlifting athletes showed the highest radial bone SOS value in the arm, whereas cycling athletes exhibited the highest tibial bone SOS value in the leg among the groups. Weightlifting athletes also had significantly higher isokinetic knee extension, shoulder extension and shoulder flexion peak torque (PT) and average power (AVG.P) (*P* < 0.05) as well as significantly greater anaerobic peak power (*P* < 0.05) compared to cyclists and squash players. However, the aerobic capacity of cyclists and squash players was significantly higher (*P* < 0.001) than the weightlifters. The cyclists had significantly higher anaerobic capacity and power (*P* < 0.001) than weightlifters and squash players.

**Conclusion:**

The findings imply that the bone health and physiological profiles of athletes are influenced by the type of sporting activity they undertake.

## Introduction

Physical activity is recognised to be beneficial for bone health and for increasing fitness (1–2). The optimum response and adaptation of bone to an applied load or strain during exercise depends on the characteristics of the strain, including the strain magnitude, rate, frequency or duration and distribution of strain stimulus (3–5). Most mechanical forces that act on the skeleton during physical activities are generated through impact with the ground and/or skeletal muscle contractions (6–8). Hence, the effects of physical activity on bone mineral density (BMD) are primarily related to the mechanisms of mechanical loading imposed on the skeleton (9). Skeletal adaptations to loading are site-specific (10–12). Bone is a highly dynamic tissue that adapts its mass and architecture to the physiological and mechanical environment (10, 13). Mechanical loading, which is a physical stress on a mechanical system, that produces high strains on specific bone sites should be repeated regularly for beneficial osteogenic effect (14–16). Mechanical loading on bones is defined as the physical stress on a mechanical system such as the bones. Lack of mechanical stress causes bones to lose minerals, collagen fibres and in turn, strength. Mechanical stress on bones can be produced by performing physical activities (e.g. running, jumping and weightlifting) that stimulate bone building or formation (15).

Weightlifting is a weight-bearing sport that involves high-intensity loading forces and requires dynamic strength and power (17). Weightlifters can create exceptionally high peak forces and contractile rates of force development during the two competition lifts, resulting in high peak power outputs and contractile impulses (18). Conroy et al. (19) reported that chronic overload experienced by elite junior weightlifters produces muscular strength that has a major influence on BMD.

Cycling is a non-weight-bearing activity that requires high-intensity effort with repeated movements. Nagle and Brooks (20) indicated that the prone position of cyclists may be inadequate to stimulate bone formation. Moreover, cycling lacks substantial impact on the skeleton due to a relatively low strain magnitude. Since only part of the body mass is used dynamically and cyclically, the physiological demands in cycling are considered to be too low to induce significant osteogenic stimulation of the bones (21–22). However, the muscle contractions associated with cycling movements during training could impose sufficient forces on the bone and subsequently promote bone health.

Squash is a weight-bearing sport that provides impact loading and involves accelerating and decelerating motions during play. The playing arm of squash players is mostly loaded by impacts during the racquet stroke in conjunction with frequent, rapid and multidirectional leg movements. Therefore, the bones of squash players are likely exposed to forces that are sufficient to produce high levels of bone formation. Squash is primarily aerobic in nature, with activity afforded from anaerobic energy sources (23) as well as a need for high levels of endurance, strength, physical agility, speed and coordination (24).

In the present study, quantitative ultrasound (QUS) measurements of the bones were carried out. QUS, a diagnostic procedure that analyses the speed of sound (SOS) in bone, is an alternative technology to DEXA scanning for determining bone density. QUS has the advantage of being radiation-free, simple to use, portable and less expensive than the DEXA scan. Bone health status can be represented by BMD.

The amount of bone loading in weightlifting, cycling and squash varies greatly. Weightlifting involves additional bone loading beyond body weight (BW). Cycling is non-weight-bearing with repetitive motions, whereas squash is weight-bearing and involves repetitive impact loading of the bone. The extent to which bony stresses from these sports affect bone health status in young male athletes in Malaysia is unclear. In addition, there are variations in the physiological demands of these three sports in terms of muscular performance and aerobic and anaerobic capacities. In this study, we investigated bone health status, muscular strength and power and, aerobic and anaerobic capacities of young Malaysian male athletes engaged in weightlifting, cycling and squash to determine the effect of different mechanical loading on the bones imposed by these activities.

## Methods

### Study Participants and Experimental Design

A total of 44 male participants aged between 15 years old and 20 years old were age-matched and then randomly assigned into four groups of 11 each: i) sedentary control; ii) weightlifting; iii) cycling and iv) squash. The study participants were healthy and had no health issues such as cancer, diabetes, asthma, stroke or heart disease. The athletes had competed in weightlifting, cycling and squash events for at least 3 years and represented Kelantan in those sports. Participants in the sedentary group were individuals who do not exercise more than twice a week. Written informed consent was obtained from the parents of the study participants who met the inclusion criteria. The study was conducted in accordance with the Declaration of Helsinki and the International Committee of Medical Journal Editors after approval by the Human Research Ethics Committee of the Universiti Sains Malaysia.

### Sample Size Calculation

The sample size used in this study was calculated using GPower software based on the measured parameter of ‘peak torque (PT)’ in a previous study carried out by Kim et al. (25). The power of the study was set at 80% with 95% confident interval, i.e. alpha at 0.05 and the standard deviation observed was 12.48 Nm. Difference in means was set at 7 Nm and the effect size was set at 0.58 for the four study groups. The total number of participants required was calculated to be 40. There were four study groups, so 10 participants were needed for each group. Considering a 10% estimated dropout rate, 11 participants were recruited for each study group.

### Body Height, Weight and Bone Speed of Sound Measurements

Body height and BW of the participants were measured using a standard stadiometer (Seca 220, Hamburg, Germany) and weighing scale (Tanita model TBF-140, Japan). The bone SOS of both upper and lower limbs of the participants was assessed at the mid-tibia shaft and radius using a bone sonometer (Sunlight Mini OmniTM, Israel) as described in previous studies (26–27).

### Muscular Performance Measurements

The participants used an isokinetic dynamometer to measure PT and AVG.P in their knee and shoulder extension and flexion muscles at two different angular velocities: 60°.s^−1^ and 300°.s^−1^ (BIODEX multi-joint system, New York), which consists of five repetitions of 60°.s^−1^ angular velocity and ten repetitions of 300°.s^−1^ angular velocity. The participants were given a 20-s rest period between each angular velocity (26–27).

### Aerobic Capacity and Wingate Anaerobic Tests

A 20-m shuttle run test was used to evaluate the predicted maximal oxygen consumption (VO_2 max_) of the participants. They were instructed to run continuously between two lines separated by 20 m in time with pre-recorded beeps. The runs were repeated until the participants could not keep up with the beeps.

The Wingate test required subjects to pedal for 30 s on a bicycle ergometer (H-300-RLode, Groningen, Holland). Mean power (MP), peak power (PP), anaerobic capacity (AC), anaerobic power (AP) and fatigue index (FI) were all measured after the test was completed.

### Statistical Analysis

Statistical analyses were performed using the Statistical Package for Social Sciences (SPSS) version 26.0 (SPSS Inc, Chicago, IL, USA). To examine significant differences in all measured parameters among the four groups, a one-way ANOVA and a Bonferroni post hoc test were used. The results of this study are reported as mean (standard deviation [SD]), with a statistical significance level set at *P* < 0.05.

## Results

### Physical and Physiological Characteristics of Study Participants

The mean age of the participants was 17.1 (SD = 1.6) years old. The mean body height of the sedentary control, weightlifting, cycling and squash groups was 164 (SD = 7.6) cm, 165.2 (SD = 5.9) cm, 165.1 (SD = 5.9) cm and 168.1 (SD = 9.8) cm, respectively. The mean BW of the sedentary control, weightlifting, cycling and squash groups was 54.0 (SD = 13.1) kg, 84.7 (SD = 23.3) kg, 51.8 (SD = 9.6) kg and 67.2 (SD = 10.1) kg, respectively. The BW of the weightlifting group was significantly higher compared to the cycling and sedentary control groups (*P* < 0.001).

**Figure 1:**
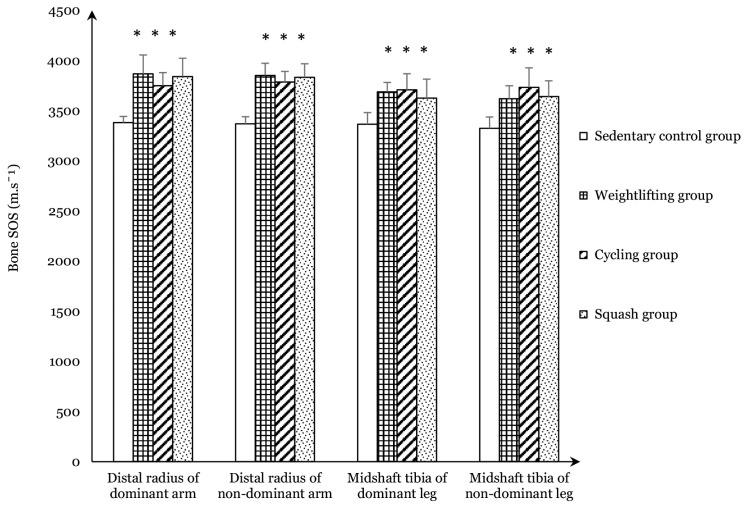
Quantitative ultrasound measurements of bone speed of sound (SOS) of dominant and non-dominant arms and legs of the participants Notes: All values are expressed as mean (SD); * = *P* < 0.05 significantly different from sedentary control group; Bone SOS = Bone speed of sound

SOS for the dominant and non-dominant upper and lower limbs of the participants was determined by measuring the mean distal radius and carrying out midshaft tibia QUS analysis of the bone. The bone SOS values for the dominant and non-dominant upper and lower limbs of the weightlifting, cycling, and squash groups were all significantly higher (*P* < 0.05) than the control group (Figure 1). The percent difference of bone SOS for the dominant arm in the weightlifting group was the highest (+14.4%), followed by squash (+13.6%) and cycling (+10.9%) groups when compared to the sedentary control group. Similarly, the percent difference of bone SOS of the non-dominant arm in the weightlifting group relative to the sedentary control group was the highest (+14.3%), followed by squash (+13.8%) and cycling (+12.4%). The percent difference of bone SOS of the dominant leg in the cycling group compared to the sedentary control group was the highest (+10.2%), followed by the weightlifting (+9.6%) and squash groups (+7.7%). The cycling group also showed the highest percent difference (+12.3%) in the non-dominant leg compared to the sedentary control, whereas the differences for the squash and weightlifting groups was +9.5% and +8.9%, respectively.

### Isokinetic Knee Extension and Flexion Peak Torque, Peak Torque Per Body Weight and Average Power

The mean values of isokinetic muscular performance of the knee at 60°.s^−1^ and 300°.s^−1^ were determined for the different groups (Tables 1 and 2). The weightlifting, cycling and squash groups had substantially higher mean values (*P* < 0.05) than the control group in most of the isokinetic measured parameters of knee extension at 60°.s^−1^ and 300°.s^−1^ for both legs. Meanwhile, weightlifting and squash athletes had higher isokinetic knee extension PT and AVG.P (*P* < 0.05) compared to the cyclists at velocities of 60°.s^−1^ and 300°.s^−1^ (Table 1).

The weightlifting, cycling and squash groups also showed significantly higher values in most measured isokinetic parameters of knee flexion at 60°.s^−1^ and 300°.s^−1^ compared to the sedentary control group (Table 2). The weightlifting and squash athletes also had higher isokinetic knee flexion PT and AVG.P (*P* < 0.05) compared to the cyclists at velocities of 60°.s^−1^ and 300°.s^−1^ Nevertheless, the cyclists showed higher knee flexion PT/BW compared to the weightlifting and squash athletes.

### Isokinetic Shoulder Extension and Flexion Peak Torque, Peak Torque Per Body Weight and Average Power

The mean values of isokinetic muscular performance of the shoulder at velocities of 60°.s^−1^ and 300°.s^−1^ were next determined (Tables 3 and 4). In all three groups of athletes, the measured shoulder extension parameters were significantly higher compared to the control group. Weightlifters had significantly (*P* < 0.05) greater shoulder extension values for PT and AVG.P at 60°.s^−1^ and 300°.s^−1^ compared to the cycling and squash athletes. The cyclists did show higher shoulder extension PT/BW compared to the squash players, however (Table 3).

In terms of shoulder flexion, significantly higher values were observed for all three athletic groups compared to the control group. Weightlifters had significantly higher shoulder extension PT and AVG.P (*P* < 0.01) at 60°.s^−1^ and 300°.s^−1^ compared to the cycling and squash group, which showed significantly higher values in shoulder flexion AVG.P compared to the cycling group (Table 4).

### Aerobic Capacity (Estimated VO_2max_) and Wingate Anaerobic Capacities

**Figure 2:**
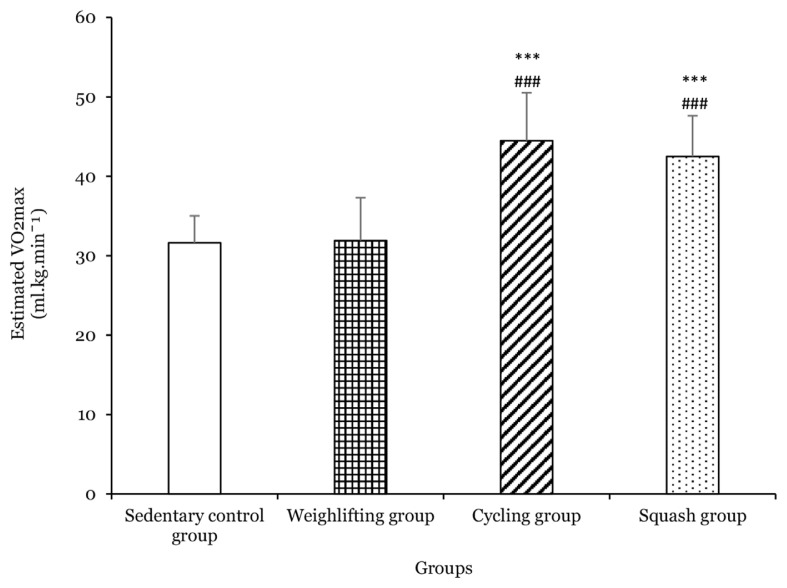
Aerobic capacity (estimated VO_2max_) of the participants Notes: All values are expressed as means ± SD; *** = *P* < 0.001 significantly different from sedentary control group; ### = *P* < 0.001 significantly different from weightlifting group

The mean aerobic capacity (estimated VO_2max_) (Figure 2) and mean Wingate anaerobic capacities (Table 5) among participants were measured. The cycling and squash groups showed significantly higher estimated VO_2max_ (*P* < 0.001) compared to the sedentary control and weightlifting groups. When compared to the cycling and squash groups, the weightlifting group had substantially higher peak power (*P* < 0.05). The cycling group exhibited significantly higher (*P* < 0.01) anaerobic capacity and anaerobic power than the weightlifting and squash groups as well as a significantly lower fatigue index (*P* < 0.05). Both weightlifting and cycling groups showed a significantly shorter time to reach peak power (*P* < 0.01) compared to the squash group.

## Discussion

Regular participation in sporting activities is known to lead to higher BMD compared to an inactive lifestyle. Bone SOS reflects the BMD of an individual (28). In the present study, we found that weightlifting, cycling and squash groups showed significantly higher values in all bone SOS of the dominant and non-dominant lower and upper limbs compared to the sedentary control group. These findings are consistent with the study done by Sagayama et al. (29), which reported that weight-classified athletes such as those who participate in wrestling and judo had higher BMD compared to nonathletes. The mechanical loading characteristics of weightlifting, cycling and squash on bones differ. Weightlifting exposes the skeleton to heavy loads in excess of normal BW, but lacks repetitive impact loading movements, whereas cycling is a non-weight bearing sport that has no direct impact loading to skeletal structures. Meanwhile, prolonged impact loading, acceleration and deceleration movements, as well as frequent changes of direction occur in squash. The findings of the present study indicate that better bone health status could be obtained by young athletes compared to sedentary controls, despite the differences in mechanical loading charateristics on the bones elicited by weightlifting, cycling and squash.

Andersen et al. (30) reported that road cycling at a national level is less effective at improving bone mass relative to weight-bearing sports. Nevertheless, our results showed that cycling training was associated with better bone health status compared to a sedentary lifestyle. This effect could be atributed to the fact that cyclists engage in prolonged training, and the repetitive muscle contraction movements during training that impose substantial force on the bones could subsequently increase bone health.

Another notable finding of the current study was that bone SOS values of both dominant and non-dominant lower limbs were highest in weightlifting group. Henriques-Neto et al. (31) noted that when athletes are subjected to high mechanical loadings on bone, site-specific adaptation of the skeleton, depending on unusual strains generated at certain sites during training by muscle stress and gravitational forces, can enhance BMD at the loaded sites. In the present study, weightlifters lifted heavy weights that induced additional bone loading beyond the BW during weightlifting training. In other words, weightlifters’ arm bones were mostly loaded with high stresses and effective strain of force. Here we indeed observed a high bone health status in the arms of weightlifters.

We also found that the SOS values of both legs of cyclists were the highest among the athlete groups. During training, cyclists’ hips are subjected to mechanical loading that is generated by high-intensity contractions of the leg and hip muscles. Impact on bone induces osteogenic stimulation at the proximal hip and femur, which would produce the high SOS values observed here for cyclists. This finding supported our hypothesis that cyclists who are involved in cycling training with prolonged duration experience repetitive muscle contractions that impose large forces on bones that subsequently produce greater improvement bone health in the legs of cyclists compared to those of weightlifters and squash players.

In terms of muscular performance, the weightlifters exhibited significantly higher knee extension, shoulder extension and flexion PT and power measurements compared to the cycling and squash groups. Kang et al. (32) found that the arm strength of weightlifters is required to maintain heavy loads above the lifter’s head for several seconds during clean and jerk competition. Kenney et al. (33) reported that muscular strength is important in weightlifting for producing maximal force to lift heavy loads during an all-out effort in the lift.

Data from the present study indicated that the strength of the upper and lower body is equally important to ensure a successful lift during competition and training in weightlifting athletes. During the two competitive lifts, the ‘snatch’ and ‘clean and jerk’, weightlifters need to generate a high peak force and fast rate of force development that consequently result in high power output (34). The lifts also require a high level of dynamic force of both the upper and lower body, with the vertebrae musculature serving as a stabiliser throughout the different phases of the lift (34). Therefore, both arm and leg strength are needed to maintain heavy loads for several seconds. Differences in movement and style could contribute to differences in knee extension, shoulder extension and flexion PT as well as power values that we observed between weightlifters and the cycling and squash athletes.

Here, squash players showed significantly higher knee extension and shoulder flexion PT and power measurements compared to the cycling group. This finding implies that higher muscular performance of the legs and arms can be achieved by prolonged squash training. These results were consistent with the study by Singh et al. (35), which found that squash players have greater strength in their knee flexors and extensors compared to non-athletes. The Singh et al.’s (35) study suggested that during squash training, muscle strength can be enhanced due to the force generated from movements having repetitive weight-bearing impact including sprinting, accelerating or decelerating movements, and frequent and rapid multidirectional leg movements.

The cyclists showed significantly higher estimated VO_2max_ compared to the weightlifting and squash groups. Cyclists make maximum use of both aerobic and anaerobic energy systems, and high aerobic capacity is particularly vital for success in cycling events (36). The findings of the present study imply that the higher aerobic capacity of the cyclists could be attributed to the nature of cycling competitions that require muscular power to be maintained at a higher percentage of VO_2max_ (37). Another factor that may contribute to the higher VO_2max_ of cyclists in the present study is that cycling events require longer training and have longer competition duration compared to weightlifting and squash matches. Thus, higher levels of VO_2max_ are critical for cyclists to sustain high levels of performance during competitions.

Our data indicated that anaerobic capacities in weightlifting, cycling and squash athletes differed. The varying physiological demands of these three sports affected the Wingate anaerobic capacities of the athletes. Notably, weightlifters exhibited significantly higher Wingate anaerobic peak power compared to cyclists and squash athletes. According to Storey and Smith (17), weightlifting requires explosive bursts of activity lasting from a few seconds to 1 to 2 minutes, and thus rely mainly on anaerobic metabolic pathways, namely the ATP-PC and glycolytic energy systems. The greater Wingate peak power of the weightlifters compared to the cyclists and squash players could be attributed to the high level of dynamic force produced by the lower body during the lifts in which weightlifters must produce high peak force and power output in a short period.

Cyclists had significantly higher Wingate anaerobic capacity and power compared to the weightlifting and squash athletes. According to Tanaka et al. (38), although the duration of cycling requires aerobic metabolism, anaerobic pathways are also involved during certain periods of cycling. Power and speed are required during the start, acceleration and final sprint. Hence, anaerobic ability is important for performance in cycling competitions since the speed of ATP re-synthesis is a highly relevant variable during critical moments when maximum and intense power is required such as in the final sprint of cycling competitions. The above explanations support observations of the present study showing that cycling athletes had higher Wingate anaerobic capacity and power values compared to weightlifting and squash athletes.

The results of the present study will expand understanding of bone health status, muscular strength and power as well as aerobic and anaerobic capacities of young male athletes in Malaysia who train regularly for sports such as weightlifting, squash and cycling. The results obtained in this study can also be applied for selecting potential weightlifting, squash and cycling athletes, and facilitate development of specific training programmes for optimal performance in weightlifting, squash and cycling.

This study has several limitations. The participants recruited were those competing at a state-level and the age range was between 15 years old and 20 years old. Moreover, only male athletes were recruited. Additional studies are needed to determine whether the findings apply to athletes in other age ranges competing at different levels. Studies involving females are also needed to determine the impact of different sports on bone health.

## Conclusion

Engagement in weightlifting, cycling and squash training could enhance bone health status compared to a sedentary lifestyle. Better bone health status of the arms was observed for weightlifters, whereas cyclists had better bone health status in the legs compared to the other groups. Isokinetic muscular knee extension, shoulder extension and flexion strength and power are important isokinetic variables in weightlifting. Weightlifters had greater Wingate anaerobic peak power compared to cyclists and squash athletes. Meanwhile, cyclists had higher aerobic and anaerobic capacities compared to weightlifters and squash players. These results imply that bone health status and physiological profiles of the athletes are dependent on the type of sports they engage in. Data from the present study is applicable in facilitating design of specific training sessions to achieve optimum performance for weightlifting, cycling and squash athletes, as well as promoting a healthy lifestyle for participants in these sports.

## Figures and Tables

**Table 1:** Isokinetic knee extension PT, PT/BW and AVG.P of the participants

			Sedentary control group (*n* = 11)	Weightlifting group (*n* = 11)	Cycling group (*n* = 11)	Squash group (*n* = 11)
60°.s^−1^	D	PT (Nm)	118.8 (20.66)	184.35 (49.86)**	140.77 (25.74)	192.8 (44.69)***,&
		PT/BW (%)	214.46 (26.57)	233.08 (73.69)	276.86 (49.97)*	291.43 (42.02)**
		AVG.P (W)	75.07 (11.82)	116.96 (29.71)**	88.39 (14.74)#	127.21 (30.23)***,&&
	ND	PT (Nm)	115.1 (26.47)	169.44 (46.8)**	137.06 (25.26)	184.65 (34.13)***,&
		PT/BW (%)	161.4 (39.61)	209.78 ( 68.82)	270.44 (56.61)***	282.12 (47.72)***,#
		AVG.P (W)	83.35 (11.18)	109.33 (30.80)*	89.96 (16.60)	116.04 (22.57)**

300°.s^−1^	D	PT (Nm)	87.76 (19.44)	123.06 (27.00)*	98.04 (22.12)	126.16 (29.00)**
		PT/BW (%)	125.65 (22.05)	156.06 (49.10)	189.71 (20.90)***	190.19 (23.30)***
		AVG.P (W)	124.35 (19.03)	278.97 (62.60)***	211.47 (48.30)**,#	277.04 (59.74)***,&
	ND	PT (Nm)	79.94 (23.04)	117.58 (38.03)*	90.87 (20.80)	117.83 (25.82)*
		PT/BW (%)	116.10 (11.73)	145.73 (53.00)	175.90 (19.80)***	178.03 (20.50)***
		AVG.P (W)	114.97 (19.10)	269.06 (94.87)***	204.76 (48.34)**	264.18 (60.80)***

Notes: All values are expressed as mean (SD);

* = *P* < 0.05;

** = *P* < 0.01;

*** = *P* < 0.001 significantly different from sedentary control group;

# = *P* < 0.05 significantly different from weightlifting group;

& = *P* < 0.05;

&& = *P* < 0.01 significantly different from cycling group;

D = Dominant limb; ND = Non-dominant limb; PT = Peak torque; PT/BW = Peak torque/Body weight; AVG.P = Average power

**Table 2:** Isokinetic knee flexion PT, PT/BW and AVG.P of the participants

			Sedentary control group (*n* = 11)	Weightlifting group (*n* = 11)	Cycling group (*n* = 11)	Squash group (*n* = 11)
60°.s^−1^	D	PT (Nm)	65.53 (19.52)	90.61 (22.46)*	76.27 (14.77)	93.97 (24.19)*
		PT/BW (%)	113.57 (20.86)	112.36 (31.1)	148.92 (20.86)**,##	141.53 ( 21.70)^#^
		AVG.P (W)	38.81 ( 9.40)	65.05 (18.90)**	56.50 (11.45)	69.36 (20.01)***
	ND	PT (Nm)	60.52 (16.8)	86.12 (32.37)	69.20 (13.86)	87.50 (21.74)*
		PT/BW (%)	110.47 (14.38)	104.95 (36.71)	134.52 (13.22)^#^	132.66 (24.48)**
		AVG.P (W)	36.88 (10.47)	57.76 (23.14)*	50.58 (10.55)	63.39 (17.19)**

300°.s^−1^	D	PT (Nm)	74.81 (15.87)	86.42 (19.17)	89.82 (14.50)	92.59 (22.90)
		PT/BW (%)	121.32 ( 9.53)	106.49 (36.33)	176.17 (22.30)***,###	140.24 (25.37)&
		AVG.P (W)	90.78 (26.88)	152.96 (46.38)**	150.23 (35.81**	158.28 (48.65)**
	ND	PT (Nm)	76.75 (25.93)	85.39 (25.80)	87.80 (15.72)	95.65 (18.18)
		PT/BW (%)	115.45 (18.84)	103.78 (42.65)	172.96 (29.78)**,###	147.33 (30.38)^#^
		AVG.P (W)	75.33 (17.63)	131.10 (56.02)*	129.96 (41.14)*	146.34 ( 31.83)**

Notes: All values are expressed as means (SD);

* = *P* < 0.05;

** = *P* < 0.01;

*** = *P* < 0.001 significantly different from sedentary control group;

## = *P* < 0.01;

### = *P* < 0.001 significantly different from weightlifting group;

& = *P* < 0.05 significantly different from cycling group;

D = Dominant limb; ND = Non-dominant limb; PT = Peak torque; PT/BW = Peak torque/Body weight; AVG.P = Average power

**Table 3:** Isokinetic shoulder extension PT, PT/BW and AVG.P of the participants

			Sedentary control group (*n* = 11)	Weightlifting group (*n* = 11)	Cycling group (*n* = 11)	Squash group (*n* = 11)
60°.s^−1^	D	PT (Nm)	50.82 (14.85)	101.12 (22.95)***	58.36 (12.98)###	75.15 (18.27)*,##
		PT/BW (%)	62.13 (23.06)	126.08 (39.96)***	114.04 (18.60)**	108.97 (29.93)**
		AVG.P (W)	34.00 (4.0)	67.35 (18.34)***	39.21 (10.61)###	52.39 (14.28)*
	ND	PT (Nm)	37.04 (10.70)	89.62 (22.00)***	52.63 (15.38)###	60.88 (15.53)*,##
		PT/BW (%)	48.87 (27.89)	109.82 (41.04)***	102.64 (23.59)**	87.75 (21.95)*
		AVG.P (W)	37.84 (4.68)	61.34 (17.28)**	36.59 (13.43)###	39.84 (12.79)##

300°.s^−1^	D	PT (Nm)	91.65 (14.27)	126.77 (16.77)***	144.35 (18.53)***	120.33 (23.08)**,^#^
		PT/BW (%)	121.64 (30.7)	181.86 (53.36)*	250.36 (42.81)***	178.36 (56.80)*,&&
		AVG.P (W)	100.25 (12.80)	182.90 (55.43)***	89.83 (35.62)###	111.94 (35.82)##
	ND	PT (Nm)	93.73 (13.79)	140.01 (17.67)***	117.15 (29.99)	111.29 (26.57)^#^
		PT/BW (%)	122.57 (32.70)	176.43 (51.06)	227.77 (54.37)***	165.95 (57.84)&
		AVG.P (W)	104.81 (4.94)	176.35 (51.26)***	77.66 (36.97)###	80.71 (39.86)###

Notes: All values are expressed as mean (SD);

* = *P* < 0.05;

** = *P* < 0.01;

*** = *P* < 0.001 significantly different from sedentary control group;

## = *P* < 0.01;

### = *P* < 0.001 significantly different from weightlifting group;

& = *P* < 0.05;

&& = *P* < 0.01 significantly different from cycling group;

D = Dominant limb; ND = Non-dominant limb; PT = Peak torque; PT/BW = Peak torque/Body weight; AVG.P = Average power

**Table 4:** Isokinetic shoulder flexion PT, PT/BW and AVG.P of the participants

			Sedentary control group (*n* = 11)	Weightlifting group (*n* = 11)	Cycling group (*n* = 11)	Squash group (*n* = 11)
60°.s^−1^	D	PT (Nm)	72.78 (11.34)	84.17 (11.17)	45.3 (12.5***,###	58.48 (15.18)###
		PT/BW (%)	106.10 (14.45)	106.46 (30.83)	88.46 (22.46)	83.65 (18.83)
		AVG.P (W)	52.12 (12.79)	61.18 (12.75)	26.08 (11.2)***,###	42.60 (12.34)&
	ND	PT (Nm)	64.85 (16.27)	81.85 (16.47)	44.31 (12.79*,###	58.96 (17.48)&
		PT/BW (%)	101.51 (20.59)	102.51 (30.98)	86.06 (17.72)	87.76 (34.77)
		AVG.P (W)	52.08 (16.53)	61.10 (16.00)	27.60 (11.17)**,###	37.91 (10.62)##

300°.s^−1^	D	PT (Nm)	95.90 (8.15)	132.74 (26.49)**	74.16 (25.59)###	94.42 (25.03)##
		PT/BW (%)	127.21 (20.59)	164.38 (41.11)	141.38 (31.22)	139.64 (49.10)
		AVG.P (W)	94.89 (13.73)	144.07 (34.85***	54.57 (25.18)**,###	87.98 (25.3)###,&
	ND	PT (Nm)	108.92 (20.10)	136.73 (23.51)	64.86 (19.48)**,###	87.05 (30.09)###
		PT/BW (%)	130.57 (17.31)	172.06 (55.45)	125.27 (30.45)	127.04 (44.53)
		AVG.P (W)	110.39 (34.41)	150.07 (40.70)*	50.4 (18.09)***,###	82.56 (28.20)###

Notes: All values are expressed as means (SD);

* = *P* < 0.05;

** = *P* < 0.01;

*** = *P* < 0.001 significantly different from sedentary control group;

## = *P* < 0.01;

### = *P* < 0.001 significantly different from weightlifting group;

& = *P* < 0.05 significantly different from cycling group;

D = Dominant limb; ND = Non-dominant limb; PT = Peak torque; PT/BW = Peak torque/Body weight; AVG.P = Average power

**Table 5:** Wingate anaerobic capacities of the participants

Variables	Sedentary control group (*n* = 11)	Weightlifting group (*n* = 11)	Cycling group (*n* = 11)	Squash group (*n* = 11)
Mean power (Watt)	326.15 (69.2)	441.68 (91.37)*	497.19 (111.72)**	498.64 (91.79)***
Peak power (Watt)	450.36 (44.82)	762.55 (93.72)***	635.00 (115.11)***,#	637.64 (105.16)***,#
Anaerobic capacity (Watt. kg^−1^)	6.59 (1.31)	5.62 (2.00)	9.58 (1.34)***,###	7.26 (1.57)&&
Anaerobic power (Watt. kg^−1^)	8.9 (0.84)	9.47 (2.12)	12.35 (1.67)*,##	9.44 (2.36)&&&
Fatigue index (Watt. s^−1^)	21.41 (3.06)	31.68 (9.00)*	13.66 (3.64)###	23.35 (10.1)&
Time to reach peak power (s)	5.41 (3.30)	6.30 (2.30)	5.71 (3.87)	13.07 (7.76)**,##,&&

Notes: All values are expressed as means (SD);

* = *P* < 0.05;

** = *P* < 0.01;

*** = *P* < 0.001 significantly different from sedentary control group;

# = *P* < 0.05;

## = *P* < 0.01;

### = *P* < 0.001 significantly different from weightlifting group;

& = *P* < 0.05;

&& = *P* < 0.01;

&&& = *P* < 0.001 significantly different from cycling group
